# Cellular mechanisms and treatments for chemobrain: insight from aging and neurodegenerative diseases

**DOI:** 10.15252/emmm.202012075

**Published:** 2020-04-29

**Authors:** Lien D Nguyen, Barbara E Ehrlich

**Affiliations:** ^1^ Department of Pharmacology and Interdepartmental Neuroscience Program Yale University New Haven CT USA

**Keywords:** aging, chemotherapy, cognitive impairment, neurodegenerative diseases, traumatic brain injury, Cancer, Neuroscience, Chemical Biology

## Abstract

Chemotherapy is a life‐saving treatment for cancer patients, but also causes long‐term cognitive impairment, or “chemobrain”, in survivors. However, several challenges, including imprecise diagnosis criteria, multiple confounding factors, and unclear and heterogeneous molecular mechanisms, impede effective investigation of preventions and treatments for chemobrain. With the rapid increase in the number of cancer survivors, chemobrain is an urgent but unmet clinical need. Here, we leverage the extensive knowledge in various fields of neuroscience to gain insights into the mechanisms for chemobrain. We start by outlining why the post‐mitotic adult brain is particularly vulnerable to chemotherapy. Next, through drawing comparisons with normal aging, Alzheimer's disease, and traumatic brain injury, we identify universal cellular mechanisms that may underlie the cognitive deficits in chemobrain. We further identify existing neurological drugs targeting these cellular mechanisms that can be repurposed as treatments for chemobrain, some of which were already shown to be effective in animal models. Finally, we briefly describe future steps to further advance our understanding of chemobrain and facilitate the development of effective preventions and treatments.

## Graphical abstract

Timely and important review on chemobrain, from the existing consensus to new potential mechanisms based on Alzheimer's disease or traumatic brain injury pathologies, suggesting chemobrain as a persistent neurological disorder.

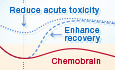

**Glossary**
Alzheimer's disease: Disease characterized by a pathological deposition of amyloid‐ß protein and fibrillary tau tangles, resulting in progressive synaptic and neuronal loss. AD initially manifests as mild short‐term memory loss, followed by severe loss of memory, speech, and executive functions.
Astrocytes: Supply neurons with oxygen, growth factors, and nutrients, and recycle ions and neurotransmitters to maintain the homeostasis of the microenvironment of the brain.
Blood–brain barrier: Forms a selective boundary between the peripheral and the central nervous systems, also blocks the entry of most drugs into the brain but can become compromised following neuroinflammation.
Chemobrain: A constellation of symptoms reflecting cognitive decline, either reversible or irreversible, that a subset of adult, non‐CNS cancer patients experience as a direct effect of chemotherapy. These effects persist even after controlling for factors such as treatment regimen, emotional status, and cancer burden.
Chemotherapy: The use of drugs to target rapidly dividing cancer cells include the alkylating agents, antimetabolites, antibiotics, topoisomerase inhibitors, and mitotic inhibitors.
Dendrites: Branching structures of neurons that receive input from other neurons.
Frontal cortex: Region important for working memory and higher cognitive functions such as planning, attention, and decision making. Loss of neurons, spines, or dendrites results in the thinning of the cortex.
Hippocampus: Structure essential for the consolidation of short‐term memory to long‐term memory, and for spatial memory.
Microglia: Resident macrophages that continuously survey the CNS for injuries and abnormalities. In response to an insult, microglia become highly motile, migrate to, and isolate the lesion site, and secrete inflammatory cytokines and phagocytose cell debris or damaged neurons.
Myelination: Formation of the insulating myelin sheath around axons, occurs rapidly during early childhood, then continues through adolescence and into adulthood.
Neurogenesis and gliogenesis: The process through which new neurons and glial cells are produced from neural precursor cells in niche regions in the brain.
Neuroinflammation: Activation of the brain's innate immunity mechanisms in response to harmful stimuli such as trauma, pathogens, and toxic metabolites.
Neurotransmitters: Chemical messengers that facilitate communications between neurons and between various brain regions.
Normal aging: Process characterized by a progressive accumulation of changes and errors at the molecular, cellular, and systemic levels, resulting in a slow deterioration of mental faculties and increased susceptibility to diseases and death.
Oligodendrocytes: Form the myelin sheath surrounding the axons of neurons, allow for faster propagation of electrical signals.
Quiescence: Cellular state characterized by reversible proliferation arrest. Dormant cells can rapidly re‐enter the cell cycle in response to various stimuli such as tissue injury.
Senescence: Cellular state characterized by irreversible proliferation arrest and increased secretion of inflammatory cytokines, growth factors, and proteases to contribute to age‐related inflammation.
Spines: Small membranous protrusions on dendrites that are specialized for making synaptic contacts.
Traumatic brain injury: Occurs when an external force injures the brain, resulting in acute symptoms such as loss of consciousness, followed by chronic symptoms

## Introduction

Cancer survival rates have significantly improved due to advances in awareness, screening, prevention, diagnosis, and treatment. For example, the average 5‐year survival rates for breast cancer increased from 75% in the 1975–1977 cohort to 91% in the 2008–2014 cohort (Noone *et al*, 2018). However, most treatments, including conventional chemotherapeutics and newer therapies such as immunotherapy, are associated with severe, sometimes long‐lasting or irreversible, side effects. With an estimate of 16.9 million cancer survivors in the United States alone in 2019 (Miller *et al*, 2019), it is clear that alleviating these side effects is an urgent clinical need.

Since the discovery of antifolates for treating acute lymphoblastic leukemia in the 1940s (Farber & Diamond, 1948), chemotherapy remains a mainstream treatment for many types of cancer (Noone *et al*, 2018), and is essential in later stages where metastasis renders local surgery insufficient. In 1978, concerns were raised about the impacts of chemotherapy on the emotional and cognitive status of cancer patients, and how they were severely underreported by clinicians and patients (Levine *et al*, 1978). However, it was not until the early 2000s that a series of epidemiological and imaging studies conclusively supported that cognitive decline in breast cancer patients had real physiological bases (Ahles & Saykin, 2001, 2002; Ahles *et al*, 2002; Saykin *et al*, 2003). Still, the underlying mechanism remains poorly understood.

We focus on the effects of chemotherapy on the central nervous system (CNS) in adults, resulting in symptoms colloquially known as chemobrain. Here, we compare chemobrain with aging, Alzheimer's disease (AD), and traumatic brain injury (TBI). We aim to leverage knowledge from more extensively studied disciplines to address the more recently acknowledged topic of chemobrain. Although disorders affecting cognitive capabilities are complex in terms of mechanisms, symptoms, risks, onsets, and anatomical loci affected, they share similarities. This review will facilitate cross‐disciplinary thinking and enable laboratories to share expertise to address chemobrain.

## Symptoms, epidemiology, and findings from imaging studies

Cognitive complaints are common among cancer patients during and after chemotherapy. Cross‐sectional and longitudinal studies suggest that short‐term memory, working memory, and verbal ability are most frequently affected, followed by visuospatial memory, executive functions, and attention span (for meta‐analyses, see Stewart *et al*, 2006; Jim *et al*, 2012; Lindner *et al*, 2014). These deficits tend to be subtle, such that cancer survivors with chemobrain perform at the lower end of the normal range, but not yet in the pathological range (Nelson & Suls, 2013). This subtlety, together with the reliance on tests designed to detect more severe, localized deficits such as TBI, strokes, and AD, means that these cognitive changes are often undetected or underestimated by clinicians (Horowitz *et al*, 2018). The difficulties with objectively defining and measuring chemobrain result in vast differences in estimating the percentage of cancer survivors with chemobrain, which range from 17 to 75% (Wefel & Schagen, 2012). Notably, the percentage of cancer survivors diagnosed with chemobrain tends to be inversely correlated with time after treatment, suggesting that some recovery occurs. Nevertheless, deficits could be detected up to 10 years after treatment, suggesting that they are permanent in some cancer survivors (Ahles *et al*, 2002).

Furthermore, structural studies reveal decreased gray matter density in several brain regions, including the frontal and temporal cortices, the cerebellum, and the right thalamus immediately after chemotherapy, with only partial recovery a year later (McDonald *et al*, 2010, 2013). Functional magnetic resonance imaging (fMRI) studies also found decreased activation during cognitive tasks in similar regions (Kesler *et al*, 2011; de Ruiter *et al*, 2011; Lopez Zunini *et al*, 2013). However, other studies found increased activation in the same regions, and propose that this is a compensatory mechanism as cancer survivors need to utilize more mental resources for the same tasks. These mental resources then become more quickly depleted for complex tasks (Ferguson *et al*, 2007; Menning *et al*, 2017). Nevertheless, together, these studies provide concrete evidence that the symptoms of chemobrain have biological bases, rather than being purely psychological.

## Chemotherapy and the post‐mitotic adult brain

**Figure 1:**
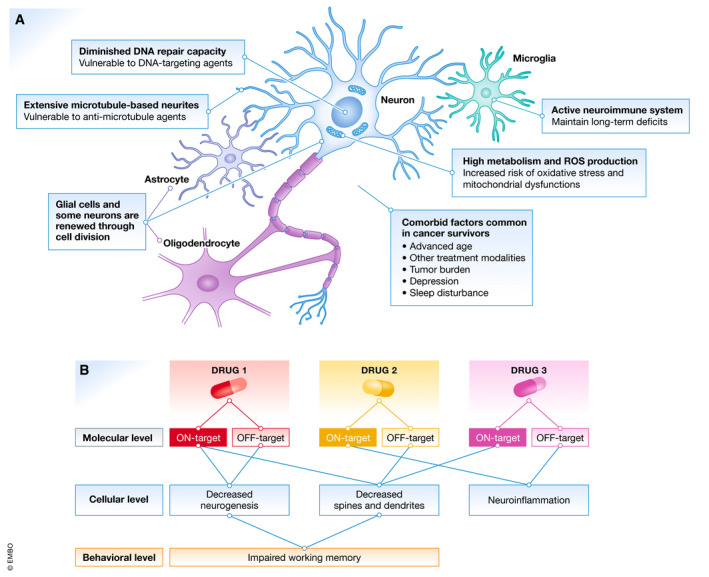
Molecular mechanisms for chemobrain are highly complex and heterogeneous (A) The central nervous system (CNS) is intrinsically vulnerable to the on‐target effects of chemotherapeutic drugs and possesses low recovery capacity. First, as most neurons are non‐dividing cells, they lack several DNA repair mechanisms that make them susceptible to DNA‐targeting agents. Second, neurons rely on an extensive microtubule‐based network for proper functions and communication, making them vulnerable to microtubule‐targeting agents. Third, chemotherapy can reduce neurogenesis and gliogenesis, which are crucial processes required for maintaining the health and plasticity of the CNS. Fourth, glial cells contribute to the vigilant neuroimmune system, and can be damaging when hyperactivated. Lastly, high metabolism, high production of reactive oxidative species (ROS), and comorbid factors common in cancer survivors make the CNS particularly vulnerable to external insults. (B) A model illustrating the complexity and heterogeneity of mechanisms for chemobrain. We propose that focusing on the cellular consequences is currently the most feasible approach for the development of treatments and preventions for chemobrain.

At first glance, cancer and neurodegeneration appear to lie on opposite ends of the disease mechanism spectrum (Plun‐Favreau *et al*, 2010). Cancer involves an abnormal resistance, whereas neurodegeneration involves an abnormal susceptibility, to cell death. Moreover, chemotherapeutic drugs are designed to selectively target rapidly dividing cells, but most neurons are non‐dividing, post‐mitotic cells, except for those in niche regions in the brain. Although several studies focus on diminished cell division, other intrinsic properties of the adult brain likely contribute to its vulnerability to chemotherapy (Fig 1A).

First, chemotherapeutics drugs, especially the DNA‐targeting agents, can cause DNA damage in post‐mitotic neurons, which accelerates senescence and eventual cell death (Hoeijmakers, 2009; Maynard *et al*, 2015). The post‐mitotic brain also exhibits diminished DNA repair capacity. Some DNA repair pathways, including mismatch repair, homologous recombination, and non‐homologous end joining, are associated with replication and therefore attenuated in non‐dividing neurons (Maynard *et al*, 2015). Thus, the accumulation of DNA damage caused by chemotherapy can accelerate neuronal dysfunction and death.

Second, many chemotherapeutic drugs target the microtubule network critical for segregating chromosomes during mitosis (Mihlon *et al*, 2010). These drugs disrupt microtubule dynamics either through hyperstabilizing (paclitaxel, docetaxel, and ixabepilone) or destabilizing (vincristine and vinblastine) microtubule formation. The microtubule network is essential for regulating neuronal polarity and morphology, axonal transport, and scaffolding signaling hubs (Dubey *et al*, 2015). Therefore, either excessive microtubule stabilization or destabilization can dysregulate neuronal morphology, functions, and communication.

Third, non‐neuronal cells, including astrocytes, oligodendrocytes, and microglia, play essential roles in maintaining the health and normal functions of the CNS. The lifelong proliferation and turnover of glial cells make them vulnerable to chemotherapy. In addition, damage to neurons or glial cells can activate microglia and astrocytes, leading to neuroinflammation that maintains chronic deficits.

Fourth, the post‐mitotic brain accounts for ~ 2% of the bodyweight, but consumes ~ 20% of glucose‐derived energy (Patel, 2016), resulting in a high production of reactive oxygen species (ROS)—a major source of DNA damage. Furthermore, a majority of cancer survivors are older, with 50% of new cases diagnosed in patients aged 55–74 (Miller *et al*, 2019). Additional stress sources, including the tumor itself and psychiatric comorbidity such as depression, contribute to a highly vulnerable brain environment. This suggests that a small insult can “tip the scale”, triggering a cascade of events resulting in chemobrain.

In addition, chemotherapeutic drugs may also have off‐target effects independent of their anticancer mechanisms. For example, our laboratory studies how paclitaxel also dysregulates calcium signaling (Boehmerle *et al*, 2006; Mo *et al*, 2012). Moreover, a typical patient receives a cocktail of drugs during chemotherapy. In this case, the molecular mechanisms for chemobrain will be a combination of (i) each drug's on‐target effects, (ii) each drug's off‐target effects, and (iii) the synergistic effects of (i) and (ii) (Fig 1B). With such complexity and heterogeneity in molecular mechanisms, potential convergent downstream cellular consequences present more readily available targets for treatments or preventions.

## Cellular mechanisms

**Figure 2:**
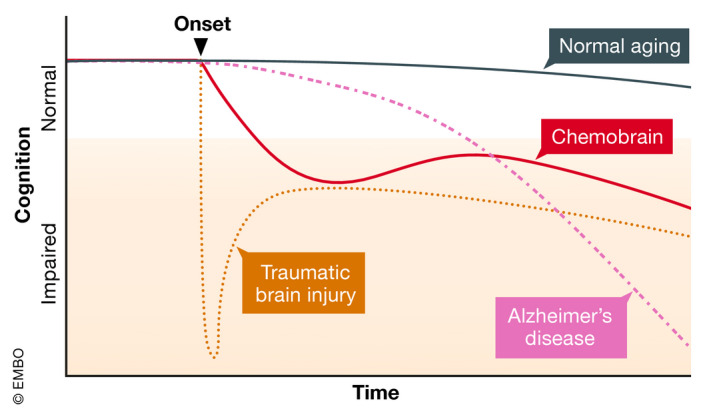
Proposed trajectory of chemobrain in comparison with normal aging, Alzheimer's disease, and traumatic brain injury Normal aging displays a slow and gradual reduction in cognitive capability over time, which, however, remains above the threshold for normal cognitive performance. Similar to Alzheimer's disease, chemobrain involves an accelerated decline in cognitive impairment, though less severe. Similar to traumatic brain injury, the onset of chemobrain is known, and the initial decline is followed by a period of recovery, which, however, may not return cognitive capability to the normal level.

In the following sections, we will draw extensive comparisons to aging, AD, and TBI due to several reasons (Fig 2). First, these conditions share similar symptoms with chemobrain, particularly impairments to memory and higher cognitive functions. Second, there is an extensive field of literature describing mechanisms and intervention strategies. Third, they represent different aspects of cognitive decline, in terms of both onset and specificity of loci affected. Similar to normal aging, chemobrain involves a subtle loss of cognitive functions, such that chemobrain has been proposed to mimic accelerated aging (Ahles *et al*, 2012), and is comparable with early stages of AD. Similar to TBI, chemobrain has a known onset, an acute phase, followed by a recovery period. Insights from aging, AD, and TBI will improve our understanding of chemobrain and facilitate the discovery of effective therapies.

### Reduced neurogenesis

After rapid cell division and maturation during the embryonic and postnatal periods, most neurons in the adult brain are fully differentiated, non‐dividing cells. Adult neurogenesis occurs primarily in niche regions: the subgranular zone (SGZ) of the dentate gyrus of the hippocampus, the subventricular zone (SVZ) lining the lateral ventricles (Ming & Song, 2011), and the striatum (Ernst *et al*, 2014). In the SGZ, neural precursor cells (NPCs) undergo cell division for self‐renewal or to give rise to immature cells that can differentiate into neurons and glial cells. At baseline, neurogenesis provides a buffer for restoring neurons lost due to daily wear and tear (Choi & Goldstein, 2018). In cases of acute insults, such as a stroke, neurogenesis after injuries is crucial for the recovery of cognitive functions (Richardson *et al*, 2007). For example, migration of cells born in the SVZ can be rerouted to injured areas such as the cerebral cortex (Sundholm‐Peters *et al*, 2005) and the striatum (Yamashita *et al*, 2006).

Reduced neurogenesis is a common factor in aging and neurodegenerative diseases. Neurogenesis declines with age, primarily through a reduction in NPCs, quiescence of the remaining NPCs, and an extracellular environment hostile to cell division (Shruster *et al*, 2010; Dubey *et al*, 2015). Neurogenesis is diminished in several AD mouse models (Hollands *et al*, 2016). Such reduced neurogenesis also increases the risk of acquiring new cognitive impairment or exacerbating existing impairment.

**Figure 3:**
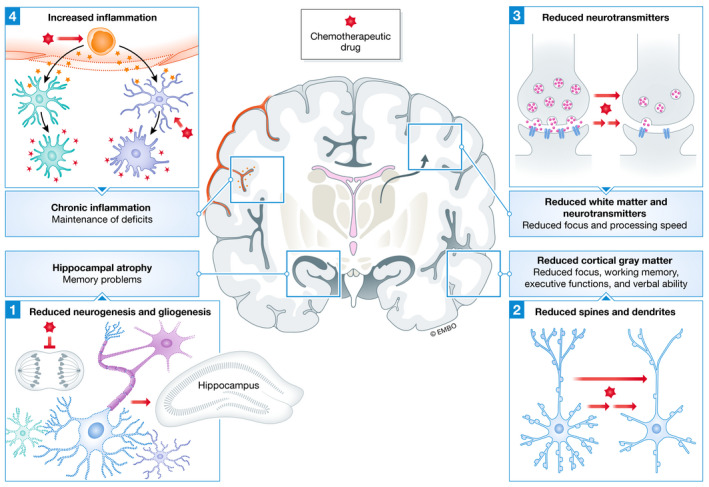
Convergent cellular mechanisms for chemobrain and how they lead to cognitive deficits The red hexagon represents a chemotherapeutic drug. First, as most drugs are designed to stop cell division, they can block neurogenesis and gliogenesis, particularly in the hippocampus. This, in turn, leads to hippocampal atrophy and memory problems. Second, chemotherapeutic drugs can lead to a decrease in cortical spines and dendrites. The subsequent loss of cortical gray matter results in impaired cortex‐based task performance, including attention, working memory, and executive functions. Third, reduced white matter due to reduced gliogenesis and alterations of neurotransmitter balance can lead to decreased focus, arousal, and processing speed. Fourth, chemotherapeutic drugs can induce peripheral or central inflammation, which hyperactivates astrocytes and microglia, resulting in chronic central inflammation that can maintain deficits for years after treatments cease.

Because memory problems are common symptoms of chemobrain, it is not surprising that reduced neurogenesis is the most commonly studied mechanism for chemobrain (Table 1 and Fig 3). Intraperitoneal injection (IP) or intravenous injections (IV) of various drugs, ranging from methotrexate, 5‐fluorouracil, cyclophosphamide, doxorubicin, docetaxel, paclitaxel, cisplatin, and thioTEPA, were observed to lead to impairment of memory from a few days to up to 20 weeks after injection (Table 1). Correspondingly, various protein markers of neurogenesis were reduced, though not in all studies. These markers include BrdU and Ki‐67, which label proliferating cells; doublecortin (DCX), which label NPCs and immature new neurons; and NeuN, which label mature neurons (Shruster *et al*, 2010). For example, several studies found a reduction in the number of BrdU‐ or Ki‐67‐positive cells, suggesting that proliferating precursor cells were directly affected (Seigers *et al*, 2008; ElBeltagy *et al*, 2010; Briones & Woods, 2011; Nokia *et al*, 2012). In contrast, 5‐fluorouracil administration did not change the number of Ki‐67‐positive cells, but caused a decrease in DCX‐positive cells (Mustafa *et al*, 2008), suggesting that the early maturation phase was affected. Similarly, cyclophosphamide or doxorubicin treatment did not change the number of BrdU‐positive cells, but reduced the number of DCX‐positive and doubly labeled BrdU/NeuN cells (Christie *et al*, 2012), suggesting that both the early and late maturation phases were affected. Future studies will benefit from assessing a range of protein markers to determine which phases of neurogenesis are affected.

**Table 1:** Summary of studies of mechanisms for development of chemobrain

Drugs and known mechanism of actions	Neurogenesis	Spines/dendrites	Neurotransmitter	Inflammation/blood–brain barrier	Glial cells
**Antimetabolites**					
Methotrexate: folate derivative, inhibits nucleotide synthesis	Seigers *et al* (2008), Lyons *et al* (2011b), Yang *et al* (2012), Wu *et al* (2017)	Wu *et al* (2017)		Yang *et al* (2012)	Seigers *et al* (2010), Geraghty *et al* (2019), Gibson *et al* (2019)
Cytarabine: pyrimidine analog, inhibits nucleotide synthesis	Dietrich *et al* (2006)				Dietrich *et al* (2006)
5‐Fluorouracil: pyrimidine analog, inhibits nucleotide synthesis	Han *et al* (2008), Mustafa *et al* (2008), ElBeltagy *et al* (2010), Lyons *et al* (2012)	Groves *et al* (2017)	Mustafa *et al* (2008), Kaplan *et al* (2016), Park *et al* (2018), Jarmolowicz *et al* (2019)	Groves *et al* (2017)	Han *et al* (2008)
**Alkylating agents**					
Cyclophosphamide: facilitates DNA crosslinks	Yang *et al* (2010), Lyons *et al* (2011a), Christie *et al* (2012)	Acharya *et al* (2015)			Christie *et al* (2012)
Cisplatin: facilitates DNA crosslinks and adducts	Dietrich *et al* (2006), Manohar *et al* (2014)	Andres *et al* (2014), Zhou *et al* (2016)			Dietrich *et al* (2006)
Carboplatin: facilitates DNA crosslinks and adducts			Kaplan *et al* (2016)		
ThioTEPA: facilitates DNA crosslinks	Mondie *et al* (2010)				
Temozolomide: methylates DNA to cause damage	Nokia *et al* (2012)				
**Mitotic inhibitors**					
Paclitaxel: binds tubulin to stabilize microtubule polymerization	Huehnchen *et al* (2017), Lee *et al* (2017)				
Docetaxel: binds tubulin to stabilize microtubule polymerization					Fardell *et al* (2014)
Vinblastine: binds tubulin to block microtubule polymerization		Parsania *et al* (2014)			
Topoisomerase inhibitors					
Doxorubicin: intercalates between DNA bases to inhibit progression of topoisomerases	Christie *et al* (2012), Park *et al* (2018)		Thomas *et al* (2017), El‐Agamy *et al* (2018), Keeney *et al* (2018)	El‐Agamy *et al* (2018), Keeney *et al* (2018)	El‐Agamy *et al* (2018)
**Combination**					
CMF (cyclophosphamide + methotrexate + 5‐fluorouracil)	Briones and Woods (2011), Rendeiro *et al* (2016)				
MF (methotrexate + 5‐fluorouracil)	Winocur *et al* (2014, 2016), Jiang *et al* (2018)				
MC (methotrexate + cytarabine)		Alexander *et al* (2018)			
AC (doxorubicin + cyclophosphamide)	Kang *et al* (2018)	Kang *et al* (2018)			
DAC (docetaxel + doxorubicin + cyclophosphamide)		Shi *et al* (2019)		Shi *et al* (2018, 2019)	

“A” refers to Adriamycin, which is the trade name for doxorubicin.

Additionally, brain‐derived neurotrophic factor (BDNF), a member of the neurotrophin family of growth factors, is secreted into the extracellular environment to promote neurogenesis. Low serum BDNF levels were associated with cognitive impairment in cancer patients (Jehn *et al*, 2015; Zimmer *et al*, 2015). In a rodent model, BDNF levels in the hippocampus decreased following injections of 5‐fluorouracil (Mustafa *et al*, 2008) or doxorubicin (Park *et al*, 2018). The mechanisms for loss of BDNF, and how this affects neurogenesis, remain unclear. However, methotrexate treatment was recently reported to deplete cortical *Bdnf* mRNA and protein expression (Geraghty *et al*, 2019), suggesting that transcriptional regulation of BDNF is an underlying factor.

While most studies focus on neurogenesis in the hippocampus, other neurogenic regions may also be vulnerable. Systemic exposure to cisplatin, cytarabine, or 5‐fluorouracil was found to decrease cell division in the SGZ, the SVZ, and the corpus callosum (Dietrich *et al*, 2006; Han *et al*, 2008). Reduced neurogenesis in multiple regions may result in symptoms beyond memory lapses. For example, in AD, olfactory dysfunction due to reduced SVZ neurogenesis is an early symptom preceding the onset of frank dementia (Zou *et al*, 2016). Furthermore, neurogenesis can be subtly affected such that no visible symptoms are observable, but survivors may still have increased risk of cognitive impairment later in life. Notably, some studies found that chemotherapy increased the risk of dementia later in life (Heck *et al*, 2008; Kesler *et al*, 2017), whereas others found no association (Baxter *et al*, 2009; Raji *et al*, 2009). Future epidemiology studies should explore these potential increased risks of neurodegenerative diseases in the population of cancer survivors compared to the control population.

### Loss of spines and dendritic arborization

Most neurons are highly polarized cells with complex morphology that are critical for their interactions and functions (Barnes & Polleux, 2009). Spines and dendrites regulate the synaptic plasticity essential for learning, memory, and executive functions (Forrest *et al*, 2018). Spines and dendrites proliferate during early development, followed by controlled pruning in childhood and adolescence, and then stabilize in adulthood. Nevertheless, both structures, particularly spines, remain dynamic in mature neurons, thereby facilitating the plasticity required for learning and adapting to new experiences (Forrest *et al*, 2018). Spines and dendrites are often reduced due to several factors, including glutamate toxicity, reduced presynaptic neurotransmitter release, protein oligomers such as amyloid‐β oligomers, unregulated calcium flux, disruption of the cytoskeleton, and disruption of the ubiquitin–proteasome system (Forrest *et al*, 2018). A gradual loss of spines and dendrites also occurs in aging (Dickstein *et al*, 2013), AD (Dorostkar *et al*, 2015), and TBI (Gao *et al*, 2011; Przekwas *et al*, 2016). These losses result in the thinning of the cortex, which may account for the reduction in gray matter in the brains of cancer survivors after chemotherapy treatment.

Several studies have observed a reduction in dendritic and spinal complexity following the administration of chemotherapeutic drugs in rodent models. Reduction in the number of spines and dendritic branching in granule cells, and CA1 and CA3 pyramidal neurons in the hippocampus, was observed following the administration of cisplatin (Andres *et al*, 2014), fluorouracil (Groves *et al*, 2017), doxorubicin, and cyclophosphamide (Acharya *et al*, 2015; Kang *et al*, 2018). In addition, reduced spine number and dendritic branching in the cingulate cortex, an integral part of the limbic system involved in emotion, learning, and memory, were observed (Zhou *et al*, 2016). Interestingly, little research has been done regarding the effect of microtubule agents, considering that the microtubule network is vital for the formation and stabilization of spines, dendrites, and axons. We are aware of only two studies linking the microtubule‐stabilizing effect of paclitaxel to impaired memory acquisition in rodent models (Atarod *et al*, 2015; You *et al*, 2018), although both studies did not further examine possible effects on neuronal morphology. There have been proposals to use these drugs to counter spine instability, specifically in AD (Brunden *et al*, 2014), supporting that the effects of microtubule agents in the CNS need to be further investigated.

Current studies are limited primarily to the hippocampus and associated regions. Future studies will benefit from examining other brain regions to determine whether the aversive effects are general or specific to particular regions. For instance, behavioral tasks to measure cortical‐based performance in chemobrain animal models, such as attention and executive functions, are lacking. We found only one study employing the 5‐choice serial reaction time task to examine prefrontal cortex impairment caused by cisplatin (Huo *et al*, 2018). Cortical‐based tasks have been developed, continuously updated, and utilized in mouse models of psychiatric disorders such as schizophrenia and bipolar disorder (Powell & Miyakawa, 2006). Similar tasks should be used to study cognitive deficits in chemobrain.

### Decreased neurotransmitter release

Neurotransmitter dysregulation, often a reduction in availability, is observed in most neurological disorders. For AD, a decrease in acetylcholine is frequently observed, which explains why three out of four FDA‐approved drugs for treating AD are acetylcholinesterase inhibitors (Graham *et al*, 2017). In aging, a loss of dopaminergic neurons, approximately 5–10% per decade, was reported (Naoi & Maruyama, 1999). The secondary injury phase of TBI is initiated by an excess of glutamate, leading to calcium overload (Walker & Tesco, 2013). Notably, a majority of neurological drugs act through modulating neurotransmitters. Prominent examples include the cholinergic system for AD, the dopaminergic system for Parkinson's disease, and the serotonergic system for depression.

Supporting evidence for the involvement of neurotransmitters comes from studies correlating variants of catechol‐*O*‐methyltransferase (COMT) with differential risks of developing chemobrain in cancer survivors. COMT regulates dopamine, epinephrine, and norepinephrine metabolism (Sheldrick *et al*, 2008). Particularly, for the COMT Val158Met polymorphism (rs4680), the Val allele is associated with higher COMT enzymatic activity, and hence lower cortical dopamine availability (Small *et al*, 2011). Consequently, cancer survivors carrying at least one Val allele are at higher risk of developing chemobrain (Small *et al*, 2011), presumably due to their smaller dopamine reservoir. Another COMT variant, rs165599 G/G, also increases the risk of chemobrain in breast cancer patients (Cheng *et al*, 2016).

Work investigating neurotransmitter alterations in chemobrain remains sparse. Mice receiving a single injection of doxorubicin had slower glutamate uptake into cells in both the cortex and the dentate gyrus (Thomas *et al*, 2017). Similarly, a reduction in dopamine release in the striatum following injections of carboplatin (Kaplan *et al*, 2016) or 5‐fluorouracil (Jarmolowicz *et al*, 2019) was reported. Serotonin release was also reduced in the raphe nucleus after carboplatin injection (Kaplan *et al*, 2016). The underlying mechanisms remain largely unclear, although reduced glutamate transporter expression (Thomas *et al*, 2017) and impaired exocytosis (Kaplan *et al*, 2016) were implicated. In addition, increased acetylcholine esterase activity was observed in the hippocampus of rats treated with doxorubicin (El‐Agamy *et al*, 2018). A reduction in choline content, the precursor for acetylcholine, was also observed after doxorubicin treatment (Keeney *et al*, 2018), suggesting that reduced cholinergic activity may be a factor in chemobrain.

Although existing research is promising, it remains unclear whether specific neurotransmitters are affected, or whether all systems are affected. As many neurological drugs target neurotransmitter systems, further studies focusing on neurotransmitters will be particularly helpful in informing therapeutic options.

### Glial cells

Glial cells are non‐neuronal cells that provide crucial support and protection for neurons, allowing neurons to perform their functions (Jessen, 2004). Similar to neurogenesis, reduced gliogenesis in the SVZ and SGZ can lead to fewer new astrocytes and oligodendrocytes. As astrocytes and oligodendrocytes modulate memory encoding and consolidation (Fields *et al*, 2014), this reduction can impair memory acquisition. Proper axonal myelination is required for fast conduction speed and enhanced cognitive processing both in rodents (McDougall *et al*, 2018) and in healthy young and elderly adults (Bendlin *et al*, 2010; Lu *et al*, 2011). Generation of new oligodendrocytes is also critical for complex motor learning (McKenzie *et al*, 2014) and spatial memory consolidation (Steadman *et al*, 2020). Imaging studies on human cancer survivors reveal a reduction in several white matter tracts (Deprez *et al*, 2011, 2012; Chen *et al*, 2020), suggesting reduced myelination. Supporting these observations, several studies reported that oligodendrocyte precursor cells (OPCs) and non‐dividing mature oligodendrocytes are especially vulnerable to chemotherapy as compared to neurons and astrocytes (Dietrich *et al*, 2006; Han *et al*, 2008; Hyrien *et al*, 2010). In addition to depleting OPCs and mature oligodendrocytes, various chemotherapeutics also alter OPC differentiation, which may further impair proper myelination (Hyrien *et al*, 2010; Gibson *et al*, 2019).

Other studies examining glial cells in neurodegenerative diseases focus on reactive gliosis, a series of events that starts with the migration of activated microglia to the site of injury, followed by activated astrocytes and oligodendrocytes, often ending with the formation of a glial scar (Burda & Sofroniew, 2014). Gliosis is the universal response to acute brain injury including TBI, ischemia, and stroke. Similarly, activated microglia and astrocytes are observed in many mouse models of AD, often predating the onset of cognitive abnormalities (Newcombe *et al*, 2018). In aging, glial cells also become activated (Lynch *et al*, 2010). These hyperactivation states and their maintenance may contribute to long‐term cognitive deficits.

Specifically, microglia activation in chemobrain occurred one week and three weeks after a single injection of methotrexate, a DNA crosslinker (Seigers *et al*, 2010). Two additional studies showed that microglia, astrocytes, and oligodendrocytes are all dysregulated following methotrexate treatment (Geraghty *et al*, 2019; Gibson *et al*, 2019). Methotrexate activates microglia, which in turn activates astrocytes, further leading to depletion of OPCs, reduced myelination, and reduced cortical BDNF levels. Astrocyte activation was observed after docetaxel injection (Fardell *et al*, 2014), and microglia activation was observed after cyclophosphamide (Christie *et al*, 2012).

The involvement of glial cells, either hypoactivation or hyperactivation, requires more investigation. As discussed in the context of other diseases such as AD, these investigations will benefit from recognizing the heterogeneity, including morphological, functional, and regional specificity, of glial cells, and whether they reduce or enhance the detrimental effects of chemotherapeutic drugs (Alibhai *et al*, 2018).

### Inflammation and breakdown of the blood–brain barrier

There is a common consensus that chronic neuroinflammation is responsible for maintaining long‐term cognitive dysfunctions in aging and neurodegenerative diseases (Glass *et al*, 2010; Michaud *et al*, 2013). Cytokines are small proteins secreted by cells of the immune system, including B cells, T cells, macrophages, mast cells, neutrophils, basophils, and eosinophils, and microglia and astrocytes (Wang *et al*, 2015a). Activated microglia and astrocytes can produce cytokines directly in CNS. However, peripherally released cytokines can also access the brain to invoke the local release of cytokines. Cytokines can also compromise the protective blood–brain barrier, thereby enabling the entrance of more cytokines and chemotherapeutic drugs (Ren *et al*, 2017). Of translational significance, peripheral and central cytokine levels can be routinely measured from the serum or the cerebrospinal fluid, making them convenient as potential biomarkers (Reale *et al*, 2009).

In aging, the gradual deterioration of the immune system, termed immunosenescence, is believed to underlie a chronic state of low‐grade inflammation (Sparkman & Johnson, 2008; Di Benedetto *et al*, 2017). AD and TBI are also associated with elevated levels of pro‐inflammatory cytokines (Remarque *et al*, 2001; Swardfager *et al*, 2010; Kumar *et al*, 2015; Schimmel *et al*, 2017). In all conditions, higher levels of inflammatory cytokines are correlated with worse cognitive performance, as well as higher morbidity and mortality (Guerreiro *et al*, 2007; Gorska‐Ciebiada *et al*, 2015; Chen *et al*, 2018). Interestingly, elevated peripheral cytokines were also observed in cancer survivors receiving various regimens of chemotherapeutic drugs (Wang *et al*, 2015a).

Few studies using animal models have examined the direct release of cytokines by activated microglia and astrocytes in the CNS. 5‐Fluorouracil and a combination of docetaxel, doxorubicin, and cyclophosphamide upregulated cytokines in the hippocampus (Groves *et al*, 2017; Shi *et al*, 2019). Methotrexate activated microglia, but no changes in CNS cytokine levels were observed (Seigers *et al*, 2010). In contrast, several chemotherapeutic drugs, including methotrexate, cisplatin, oxaliplatin, paclitaxel, and vincristine, elevated peripheral inflammatory cytokines to induce chronic pain (Brandolini *et al*, 2019). Elevation of peripheral cytokines may also penetrate the blood–brain barrier to directly act on the CNS, and to activate microglia and astrocytes to secrete further cytokines. However, the correlation between elevated peripheral cytokines and their effect on CNS inflammation remains poorly understood.

## Therapeutic avenues: current status, challenges, and repurposing existing drugs

Despite significant advances in our understanding of chemobrain, both at the clinical level and at the cellular–molecular basis, several challenges persist. First, despite increased awareness, there are currently no validated or approved tests for the diagnosis of chemobrain. Indeed, many studies find that cancer survivors score within, albeit often at the lower end of, the normal range of the population (Horowitz *et al*, 2018). This limitation is likely due to the lack of sensitivity of assessment tools used (Horowitz *et al*, 2018). Second, chemobrain is highly heterogeneous, with many confounding factors including genetic variability, treatment regimen, and comorbidity with other neurological conditions. Third, there are no clear disease mechanisms for chemobrain. Each chemotherapy drug is expected to exert a range of effects, which further vary when combined with other drugs and treatment modalities. Owing to the complexity and unclear mechanisms, the current clinical approach is to refer cancer survivors to psychiatrists who can prescribe cognitive rehabilitation, changes to lifestyle, mind‐training exercises, cognitive–behavioral therapy, and general coping strategies (Ferguson *et al*, 2012; Kesler *et al*, 2013; Henneghan & Harrison, 2015). Additionally, neuropsychiatric drugs may be prescribed to alleviate symptoms (Vance *et al*, 2017).

**Figure 4:**
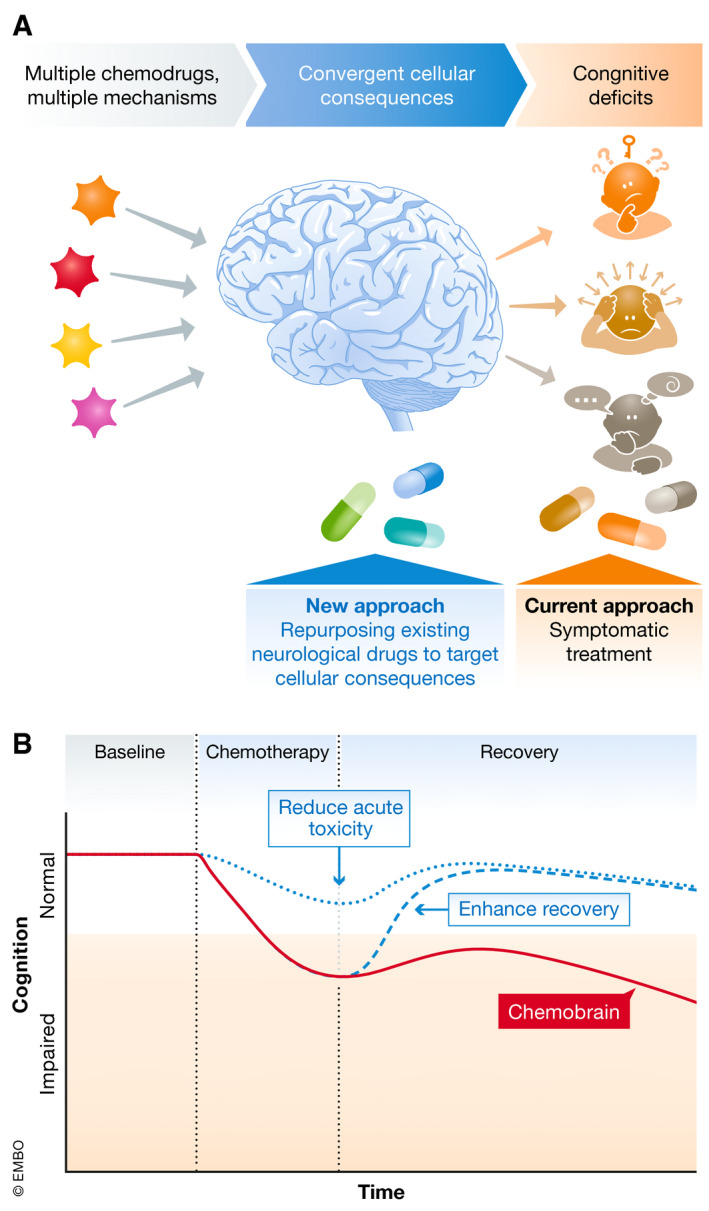
Re‐purposing of existing approved drugs to treat chemobrain (A) Although the current clinical approach is to prescribe interventions to treat the behavioral symptoms of chemobrain, a more targeted approach is to prescribe interventions that address likely convergent cellular consequences such as those discussed in “Cellular mechanisms”. (B) With sufficient knowledge of both cellular and molecular mechanisms, we can aim to directly modify the trajectory of chemobrain, either through reducing acute toxicity during chemotherapy, or enhancing recovery after chemotherapy, to return cognitive capability to the normal level.

Considering the complexity of discovering, fine‐tuning, and approving new therapeutic compounds for the CNS (Pangalos *et al*, 2007), we propose that repurposing existing drugs is a feasible approach to successfully treating chemobrain in the near future. Despite the heterogeneity of molecular mechanisms, there are convergent cellular mechanisms that can be targeted (Table 2 and Fig 4A). This, of course, is not to discount the importance of studies that continue to examine the specific molecular mechanisms of each chemotherapeutic drug. With sufficient knowledge of the consequence of chemotherapy at all levels—molecular, cellular, and behavioral—better prevention or treatment options can be developed. Eventually, the more efficient therapies will not only treat the symptoms but also directly modify the trajectory of chemobrain (Fig 4B), either through alleviating the initial toxic effects or through enhancing recovery after chemotherapy.

**Table 2:** Therapeutic strategies for preventing or alleviating chemobrain

Cellular mechanism	Potential therapeutic options	
	Tested in models of chemobrain	Tested in models of aging and neurodegenerative diseases
Reduction in neurogenesis and gliogenesis	Exercise (Fardell *et al*, 2012; Winocur *et al*, 2014; Park *et al*, 2018)	Neurotrophic factors: BDNF, GDNF, NGF, VEGF, IGF‐1
	Environmental enrichment (Winocur *et al*, 2016)	Transcranial magnetic stimulation
	Lithium (Huehnchen *et al*, 2017)	
	SSRIs: fluoxetine (ElBeltagy *et al*, 2010; Lyons *et al*, 2011b, 2012)	
	Stem cell transplantation (Acharya *et al*, 2015)	
Loss of spines and dendritic structure	Metformin (Zhou *et al*, 2016)	Neurotrophic factors
	PDEIs: rolipram (Callaghan & O'Mara, 2015), ibudilast (Johnston *et al*, 2017)	Other PDEIs: sildenafil, roflumilast, milrinone, cilostazol, tadalafil
Reduction in neurotransmitter release	ACheIs: donepezil (Winocur *et al*, 2011; Lim *et al*, 2016) and astaxanthin (El‐Agamy *et al*, 2018)	Other ACheIs: tacrine, rivastigmine, galantamine
	NMDAR antagonists: dextromethorphan (Vijayanathan *et al*, 2011) and memantine (Cheng *et al*, 2017)	Dopamine and norepinephrine modulators: amphetamines, atomoxetine, methylphenidate, bupropion
		Glutamate modulators: riluzole, ketamine
Glial cells	Microglia inhibitor/depletion: PLX5622 (Gibson *et al*, 2019)	Microglia inhibitors: minocycline
	Rescue oligodendrocyte and myelination: LM22A‐4 (Geraghty *et al*, 2019)	Oligodendrocyte precursor cell transplantation
Inflammation and blood–brain barrier breakdown		NSAIDs: aspirin, ibuprofen
		Immunosuppressant drugs: copaxone, rituximab, and cladribine
		Monoclonal antibodies: anti‐TNF, anti‐IL‐1, anti‐IL‐6

ACheIs, acetylcholinesterase inhibitors; BDNF, brain‐derived neurotrophic factor; GDNF, glia‐derived neurotrophic factor; IGF‐1, insulin‐like growth factor 1; IL, interleukin; NGF, nerve growth factor; NMDAR, *N*‐methyl‐d‐aspartate receptor; NSAIDs, non‐steroidal anti‐inflammatory drugs; PDEIs, phosphodiesterase inhibitors; SSRIs, selective serotonin reuptake inhibitors; TNF, tumor necrosis factor; VEGF, vascular endothelial growth factor.

### Targeting neurogenesis

The adult hippocampus remains plastic and sensitive to environmental changes, and is therefore highly amenable to treatments (Ming & Song, 2011). Physical exercise and environmental enrichment enhance neurogenesis and alleviate symptoms in rodent models of aging (Voss *et al*, 2013), AD (Lazarov *et al*, 2010), TBI (Bondi *et al*, 2014), depression (Samuels & Hen, 2011), and chemobrain (Fardell *et al*, 2012; Winocur *et al*, 2014, 2016; Park *et al*, 2018). BDNF secretion is also essential for maintaining proper spines and dendrites, and for promoting neurogenesis in the hippocampus. BDNF secretion was increased by exercise in animal models (Lima Giacobbo *et al*, 2019). Additionally, electroconvulsive shock treatment and deep brain stimulation, often used for treating major depression, are also effective through enhancing neurogenesis (Schoenfeld & Cameron, 2015). However, studies examining the efficiency of these electrical treatments for ameliorating symptoms of chemobrain are lacking.

Many classical antidepressant drugs, including fluoxetine, reboxetine, and tranylcypromine, increase neurogenesis in the adult hippocampus (Schoenfeld & Cameron, 2015; Shohayeb *et al*, 2018). Lithium, a mood stabilizer used to treat bipolar disorder and complement treatments for depression, also improves neurogenesis (Young, 2009). Notably, fluoxetine and lithium reduce cognitive impairment in rodent models of chemobrain (ElBeltagy *et al*, 2010; Lyons *et al*, 2011b, 2012; Huehnchen *et al*, 2017). Therefore, antidepressant drugs may be useful in addressing both the cellular deficits and the behavioral manifestations of chemobrain.

Transplant of neural stem cells into various brain regions, including the hippocampus, frontal cortex, and striatum, is currently intensively studied as an approach to replace lost neurons in neurodegenerative diseases (Lindvall & Kokaia, 2010). Cells, either of human or of rodent origins, injected into rodent models, successfully survive, integrate, and differentiate into neurons and glia in the recipient's brain, and alleviate cognitive impairment (Wang *et al*, 2015b). One study found that transplantation of human neural stem cells into the hippocampus of rats rescued behavioral and cellular deficits caused by cyclophosphamide (Acharya *et al*, 2015), suggesting that this is a promising, albeit very invasive, approach.

### Targeting spines and dendrites

Spine formation and stabilization also remain highly dynamic and sensitive to environmental changes in adulthood (Forrest *et al*, 2018). The glutamate receptors, particularly the *N*‐methyl‐d‐aspartate receptors (NMDARs) and α‐amino‐3‐hydroxy‐5‐methyl‐4‐isoxazolepropionic acid receptors (AMPARs), play critical roles in spine formation and stabilization. For example, memantine, an NMDAR inhibitor, and dextromethorphan, a non‐competitive NMDAR antagonist, rescued cognitive impairment induced by cisplatin and methotrexate, respectively (Vijayanathan *et al*, 2011; Cheng *et al*, 2017). Other regulators of the NDMARs and AMPARs, including ketamine and the benzamides, can induce spine formation or remodel the dendritic arborization to reverse symptoms of depression and alleviate cognitive impairment (Partin, 2015; Phoumthipphavong *et al*, 2016; Duman, 2018). Although reversing a reduction in spines would be the desired outcome in the context of chemobrain, glutamate modulators will need careful investigation. Special attention is warranted because glutamate overload, as often is the case in TBI, can damage neurons, and reduce spinal and dendritic complexity.

Calcium signaling is also important for the proper maintenance of spines and dendrites (Higley & Sabatini, 2012). For example, dysregulated calcium/cyclic adenosine monophosphate (cAMP) signaling, such as during stress, can lead to spine destabilization and loss (Arnsten, 2015). Interestingly, phosphodiesterase inhibitors, which regulate cAMP levels, including rolipram and ibudilast, rescued cognitive impairment induced by docetaxel and oxaliplatin, respectively (Callaghan & O'Mara, 2015; Johnston *et al*, 2017). Calcium can also activate several protein kinase C isoforms, which in turn phosphorylate and activate myristoylated alanine‐rich C‐kinase substrate (MARCKS), an important regulator of spinal and dendritic complexity. Hyperactivation of protein kinase C underlies the reduction in dendritic complexity and cognitive impairment in aging and chronic psychological stress (Hains *et al*, 2009; Brudvig & Weimer, 2015), suggesting that inhibition of protein kinase C is a promising therapeutic strategy.

### Targeting neurotransmitters

Because the homeostasis of various neurotransmitters is required for normal cognitive performance (Noudoost & Moore, 2011), a majority of drugs approved for treating neurological disorders are regulators of neurotransmitters. These drugs include the selective serotonin reuptake inhibitors (SSRIs) and the acetylcholinesterase inhibitors (tacrine, donepezil, rivastigmine, and galantamine), which have been approved to treat depression and AD, respectively. Donepezil alleviated cognitive problems in two studies of chemobrain (Winocur *et al*, 2011; Lim *et al*, 2016).

Catecholaminergic drugs, which are used to treat ADHD, may also help with the attention deficits associated with chemobrain. Examples include bupropion, a dopamine reuptake inhibitor; atomoxetine, a norepinephrine reuptake inhibitor; and amphetamine and methylphenidate, which enhance both dopamine and norepinephrine availability (Heal *et al*, 2012). Bupropion and methylphenidate reduced cancer‐related (including chemotherapy) fatigue (Cullum *et al*, 2004; Gong *et al*, 2014), although methylphenidate had no effect on depression and cognition. Animal studies would provide mechanisms to complement the results of findings in human patients.

### Targeting neuroinflammation and glial cells

Neuroinflammation remains a significant risk factor for neurodegeneration and can be targeted at both the peripheral and central levels. Several large‐scale studies have examined the effects of over‐the‐counter non‐steroidal anti‐inflammatory drugs (NSAIDs) such as aspirin, ibuprofen, and naproxen on preventing or treating AD. However, the results are highly mixed, ranging from beneficial, to neutral, to harmful (Ozben & Ozben, 2019). Drugs approved for treating multiple sclerosis, a disease characterized by excessive inflammation and blood–brain barrier disruption, work through actively suppressing the immune system. Examples include copaxone, rituximab, and cladribine, which target T cells and B cells, and natalizumab, which blocks the migration of immune cells across the blood–brain barrier (Gholamzad *et al*, 2019). Because these drugs are associated with serious side effects including systemic immunosuppression and liver damage, the risks may outweigh the benefits of reducing mild cognitive impairment in chemobrain.

Neuroinflammation can also be targeted by directly targeting astrocytes and microglia. Recent evidence shows that PLX5622, a colony‐stimulating factor 1 receptor (CSF1R) inhibitor that specifically eliminates microglia, could block methotrexate‐induced memory deficits (Gibson *et al*, 2019). PLX5622 also reduced inflammation and rescued cognitive deficits in a mouse model of AD (Dagher *et al*, 2015). Minocycline is a common antibiotic drug that also inhibits microglial activation (Kobayashi *et al*, 2013). However, findings about minocycline's effects in animal models of AD and TBI have been mixed, ranging from beneficial to harmful (Garwood *et al*, 2010; Ferretti *et al*, 2012; Yang *et al*, 2012; Scott *et al*, 2018). Minocycline also did not delay the progression of cognitive impairment in people with mild AD over a 2‐year period (Howard *et al*, 2019). These results suggest that more specific targets of microglia or astrocytes are required to alleviate cognitive impairment without also triggering side effects.

As white matter tracts are often compromised following chemotherapy (Matsos *et al*, 2017), improving oligodendrogenesis and myelination is another therapeutic strategy. LM22A‐4, a TrkB agonist that promotes OPC proliferation and oligodendrogenesis, was found to rescue methotrexate‐induced myelin loss and cognitive impairment (Geraghty *et al*, 2019). In addition, OPC transplantation has long been investigated as a treatment for demyelinating diseases such as multiple sclerosis (Ben‐Hur, 2011) and, more recently, for spinal cord injury (Assinck *et al*, 2017), and can be repurposed for treating chemobrain.

## Future directions

In recent years, chemobrain has gained attention as a serious side effect of chemotherapy, and several studies have advanced our understanding of the underlying mechanisms and factors. Going forward, addressing chemobrain will require concerted efforts on multiple fronts, informed by similar efforts made for aging, AD, and TBI (Langa & Levine, 2014). On the clinical front, efforts are needed to raise awareness about the risk of chemobrain among clinicians, chemotherapy patients, and their caretakers, thereby enabling more vigilant lookout for subtle deficits such as memory lapses that may otherwise be overlooked. Improvement in sensitivity of diagnostic tools to detect mild cognitive impairment, as well as utilization of neuroimaging techniques, such as structural brain MRI for possible hippocampal atrophy, and positron‐emission tomography (PET) imaging for hypometabolism, will improve the sensitivity and reliability of chemobrain diagnoses. In addition, epidemiology studies will continue to determine whether genetic risk factors for neurodegenerative diseases, for example, variations in apolipoprotein E (APOE) (Ahles *et al*, 2003; Mandelblatt *et al*, 2018) or COMT, can predict risks of developing chemobrain in cancer survivors. Conversely, cancer survivors who do not show symptoms of chemobrain immediately after treatment may also be at increased risks of developing neurodegenerative diseases later in life. On the basic science front, efforts are needed to establish animal models that better mimic the complexity and subtlety of chemobrain in humans. Examples include utilizing animal models that are aged or carry tumors and that receive common combination of drugs instead of a single drug. In addition to tasks measuring memory acquisition, tasks that measure working memory, attention, and executive functions are also needed in studying chemobrain. Additionally, determination of whether specific cognitive modalities, anatomical regions, or cell populations are more vulnerable will further aid efforts to develop therapeutic compounds. With these focused approaches, the future for improved therapies is promising.

## Conflict of interest

B.E.E is a founder of Osmol Therapeutics, a company that is targeting neuronal calcium sensor 1 for therapeutic purposes, including chemotherapy‐induced neuropathy.

**Pending issues**

(1) Refinement of diagnosis criteria for chemobrain, including utilization of diagnostic imaging tools.
(2) Investigation of genetic risks and biomarkers for chemobrain, and whether cancer survivors are at increased risk of neurodegenerative diseases later in life.
(3) Development of animal models that better capture the complexity of chemobrain, including animals of single and combinatorial chemotherapy drugs and potential rescue with antichemobrain drugs.
(4) Determination of whether specific cognitive modalities, anatomical regions, and cell populations are more vulnerable to chemotherapy.

### For more information

https://www.cancer.gov/about-cancer/treatment/research/understanding-chemobrain

https://www.mayoclinic.org/diseases-conditions/chemo-brain/symptoms-causes/syc-20351060

https://www.cancer.org/treatment/treatments-and-side-effects/physical-side-effects/changes-in-mood-or-thinking/chemo-brain.html

https://www.mdanderson.org/patients-family/diagnosis-treatment/emotional-physical-effects/chemobrain.html
